# Exsanguination from an arteriovenous dialysis fistula: accident, suicide or medical malpractice?

**DOI:** 10.1007/s12024-025-00955-3

**Published:** 2025-02-24

**Authors:** S. Schof, J. Hertzberg, A. Jahnke, Christoph G. Birngruber

**Affiliations:** Institute of Legal Medicine, University Hospital Frankfurt, Goethe-University, Kennedyallee 104, 60596 Frankfurt am Main, Germany

**Keywords:** Exsanguination, Dialysis, Complication, Arteriovenous shunt, Renal insufficiency

## Abstract

A female senior dialysis patient was found dead in her apartment, covered in blood. Bloodstains were observed in different rooms of the apartment. During the post-mortem examination on site, a small, roundish opening of the skin was observed on the flexor side of the upper arm, within a longitudinal scar, from which blood was draining. Throughout police investigation, the possibility of an accident, a suicidal act, or medical malpractice during dialysis care was considered. An autopsy was ordered for further clarification. The autopsy identified exsanguination from a fistula on the flexor side of the left upper arm as the cause of death. The fistula could be traced into an arteriovenous shunt vessel that had been created a long time ago for dialysis. Upon projection onto the shunt vessel, punctiform crusts with underlying hemorrhages in the subcutaneous fatty tissue were identified in the skin. Histological examinations of the fistula and its surrounding tissue revealed no evidence of vasculitis or perivascular inflammatory changes, but puncture sites of varying ages with connective tissue texture disruption of the vessel wall and the adjacent subcutaneous tissue. Forensic medical examination concluded that death was caused by bleeding from an arteriovenous dialysis shunt vessel as a complication of hemodialysis. This case illustrates the relevance of comprehensive forensic medical case processing as the basis for a well-founded assessment.

## Introduction

Worldwide, approximately 850 million individuals are afflicted with chronic kidney failure [1] of whom 3.9 million are reliant on hemodialysis [2].

An arteriovenous fistula (AVF), or dialysis shunt, is the best established dialysis technique for a long term dialysis treatment. However, it entails a number of potential complications, including aneurysms, infections, vascular steal syndrome, and bleeding [3–5]. Although fatalities resulting from bleeding are a common occurrence in forensic autopsies [6], cases of exsanguination from a dialysis shunt are exceedingly rare [5].

For a proper assessment to be made, it is important to clarify whether the bleeding could be the result of an accident, a secondary effect of some underlying medical condition, an unintended consequence of a therapeutic procedure, or a suicidal act [7, 8].

This article presents the case of a senior dialysis patient who bled to death from an arteriovenous shunt vessel. Questions and problems that arise for forensic medical casework are discussed.

## Case report

### History and site

A 73-year-old woman did not attend her scheduled dialysis appointment. Subsequently, the daughter went to her mother’s apartment and discovered her body in a pool of blood in the bathroom. The emergency services pronounced her dead at the scene and called the police due to the large amount of blood in different rooms of the apartment. In the bedroom, numerous open medication packs, scissors and bloody bandages were found. A home emergency call system in the apartment had not been used. The police considered the involvement of third parties to be possible and ordered a forensic external post-mortem examination at the scene to further clarify the circumstances of death.

### External post-mortem examination at the site

During the external post-mortem examination, a small, roundish skin opening was found on the flexor side of the left upper arm, within a longitudinal scar, from which blood was draining. No other injuries were evident. A notable decrease in livor mortis was observed.

**Fig. 1:**
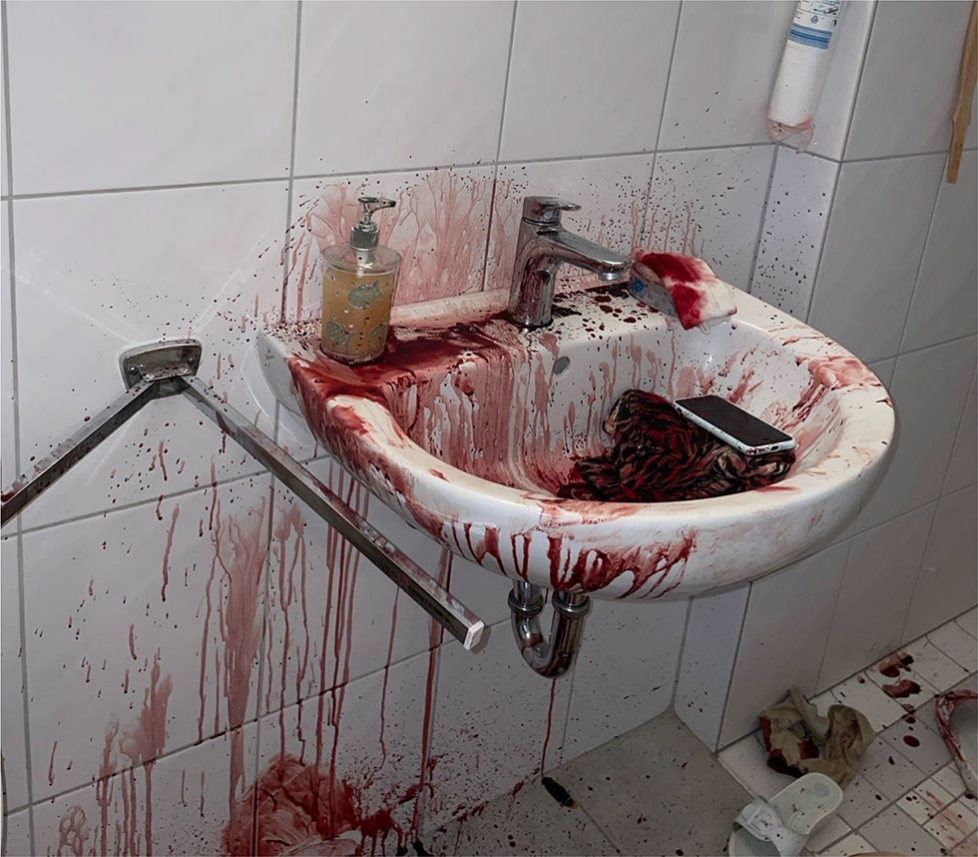
The bloodstain pattern in the bathroom suggests bleeding from the high-pressure system

The traces of blood in the apartment consisted mainly of blood drop marks on the floor and pools of blood in various places. In the bathroom, evidence of bleeding from the high-pressure system was observed (Fig. 1), as well as a substantial quantity of blood. The overall distribution pattern of the bloodstains seemed calm and consistent with the hypothesis that a person experiencing significant bleeding had walked through the apartment.

The findings at the scene and on the corpse suggested a hemorrhage from an injury on the left upper arm, presumably from a shunt vessel. As the underlying cause remained unclear, an autopsy was recommended.

### Further police investigation

Police investigation revealed that the woman had her last dialysis appointment three days before and had last been seen alive the day before her body was found.

Several decades ago, she underwent nephrectomy. Due to nephrosclerosis of the remaining kidney, a kidney transplant was necessary. After a transplant failure, she developed terminal kidney failure, whereupon she required outpatient hemodialysis three times a week for about 10 years. As a result, she suffered of secondary hyperparathyreodism and renal anemia. Despite her troubled state of health, neither her family nor her doctors reported any suicidal tendencies.

At the dialysis facility, the police were informed that puncture conditions were challenging due to metabolic syndrome with class III obesity and type 2 diabetes mellitus. The shunt vessel ran predominantly deep in the soft tissue and has been operated on several times, which is why the dialysis access could only be placed on a short section of the vessel. Various other access methods (cervical, brachial) had been attempted in the past and repeatedly led to vascular occlusions.

The police wondered whether the shunt had been adequately treated after the dialysis and whether medical malpractice could have been a contributing factor to the death. An autopsy was ordered.

### Autopsy

The autopsy was performed three days after the body was found.

The body weight was 98 kg, the height was 150 cm. A fistula-like skin opening measuring approx. 0.3 cm in diameter with a raised gray-white border wall was found within a long, old surgical scar on the flexor side of the left upper arm. Two small scabs were visible next to the skin opening. The surrounding skin exhibited a blue-red, mottled discoloration, with a suggestive rectangular shape (Fig. 2a). Dissection of the upper arm revealed a fistula-like connection between the skin opening and an arteriovenous shunt vessel running underneath it. This vessel exhibited an aneurysmal bulge measuring 4 cm in circumference and isolated arteriosclerotic changes (Fig. 2b). The subcutaneous fatty tissue showed a slight black-red hemorrhage.

**Fig. 2a:**
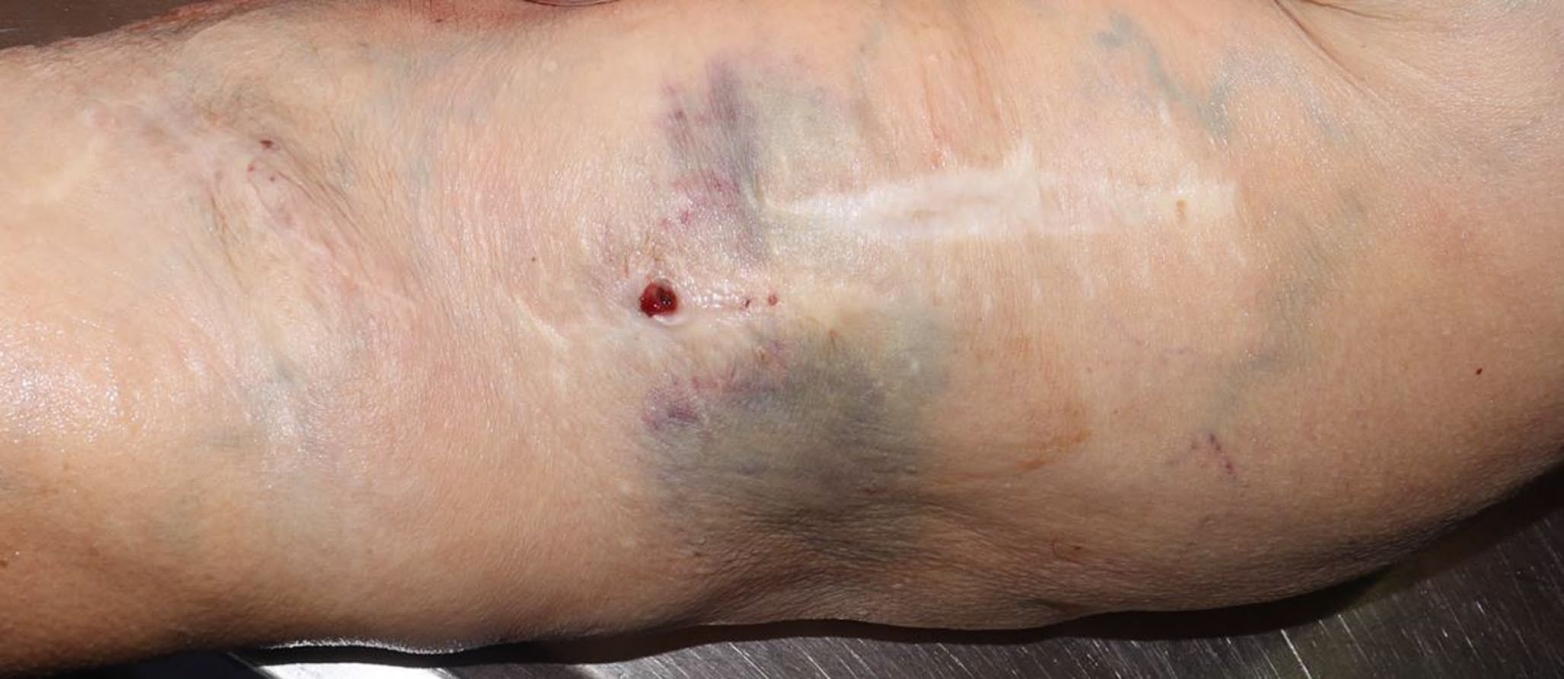
Fistula-like skin opening within the surgical scar with connection to the shunt vessel running underneath, surrounding skin discoloration on the left upper arm

**Fig. 2b:**
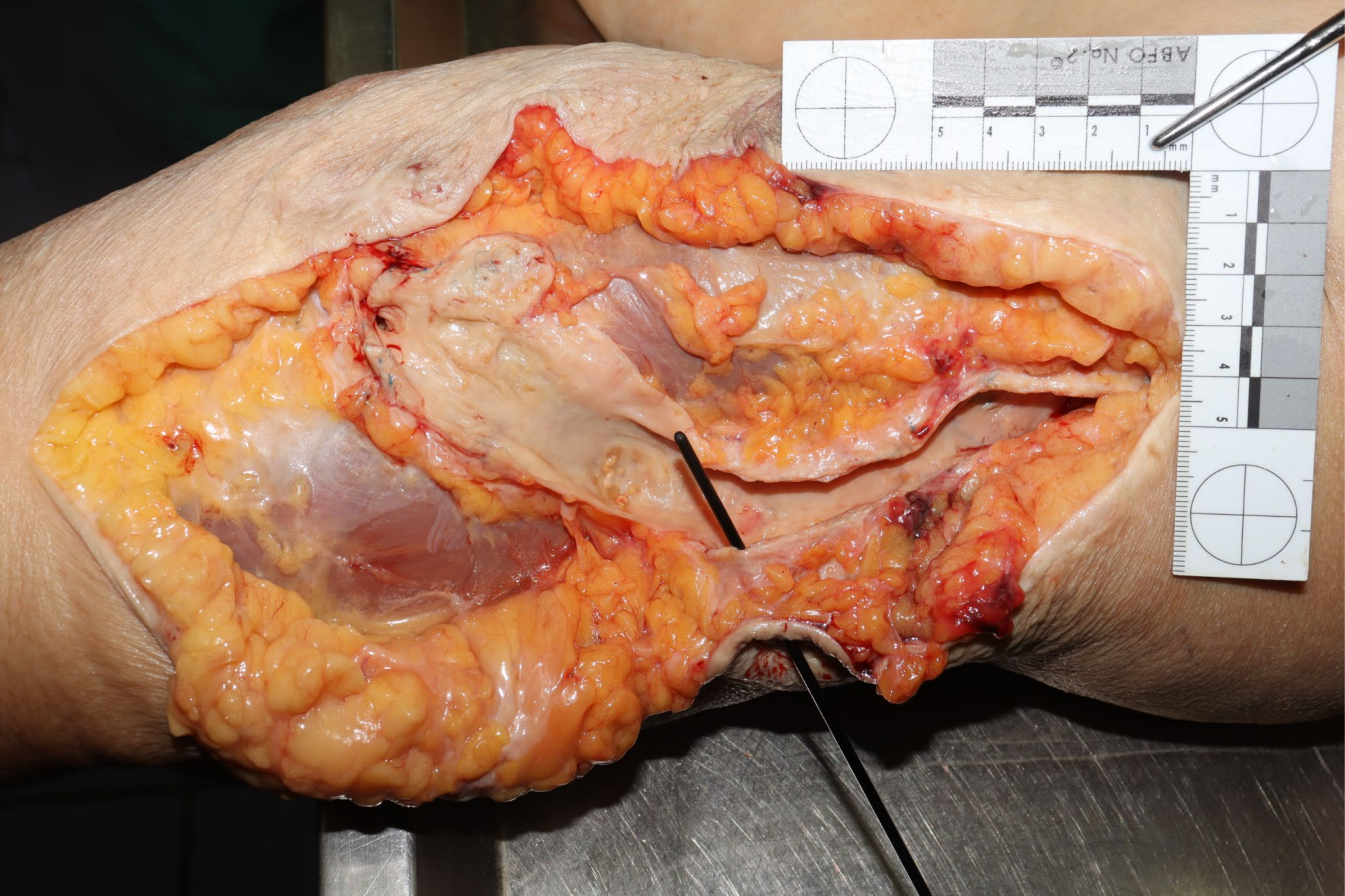
Shunt vessel after dissection, the probe is inserted into the fistula

Livor mortis and the blood content of the internal organs were clearly reduced. The spleen capsule was wrinkled, and the endocardium of the left ventricular outflow tract displayed streaky hemorrhage. The existing autologous kidney showed scarred retractions and significant reduction in functional tissue, accompanied by a lack of medullary-cortical differentiation. The liver was fatty and cirrhotic and there were foci of hematopoiesis on the skull cap.

The autopsy revealed no evidence of external violence by a third hand. The cause of death was exsanguination from a fistula in the dialysis shunt.

### Histology

Histological examinations revealed mainly age-appropriate changes. Remarkable abnormalities were observed in the kidney, liver and in the soft tissues around the shunt vessel.

#### Kidney

**Fig. 3:**
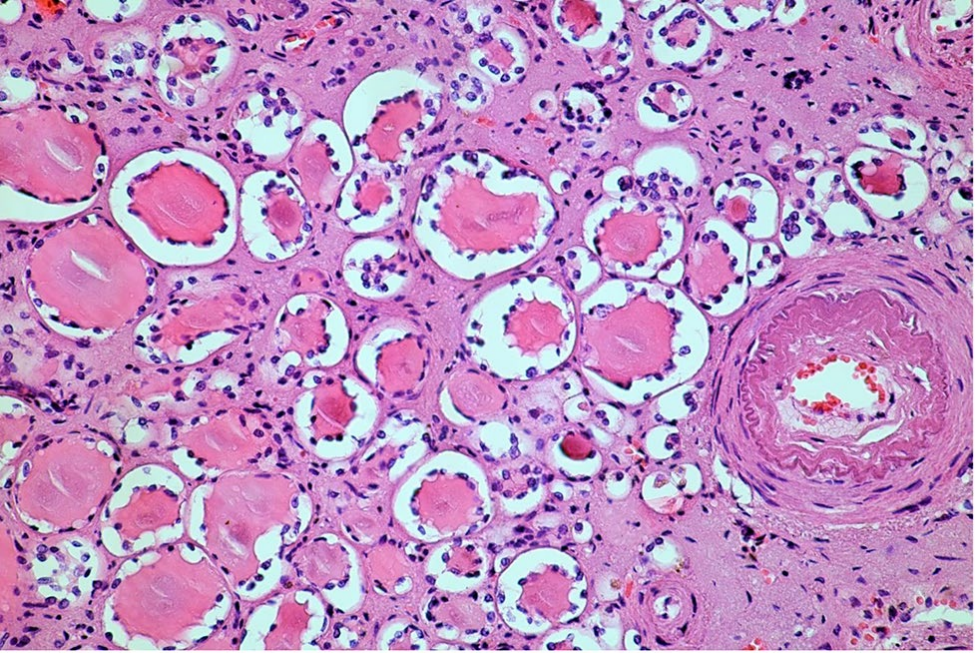
Strumigenic pattern of the pyelonephritic shrivelled kidney (HE, magnification 200:1)

There was a “strumigenic transformation” of the renal functional tissue (Fig. 3) with almost complete tubular atrophy. Renal corpuscles could only be guessed at and were predominantly sclerosed. The clinically reduced functionality was very well comprehensible.

#### Liver

**Fig. 4:**
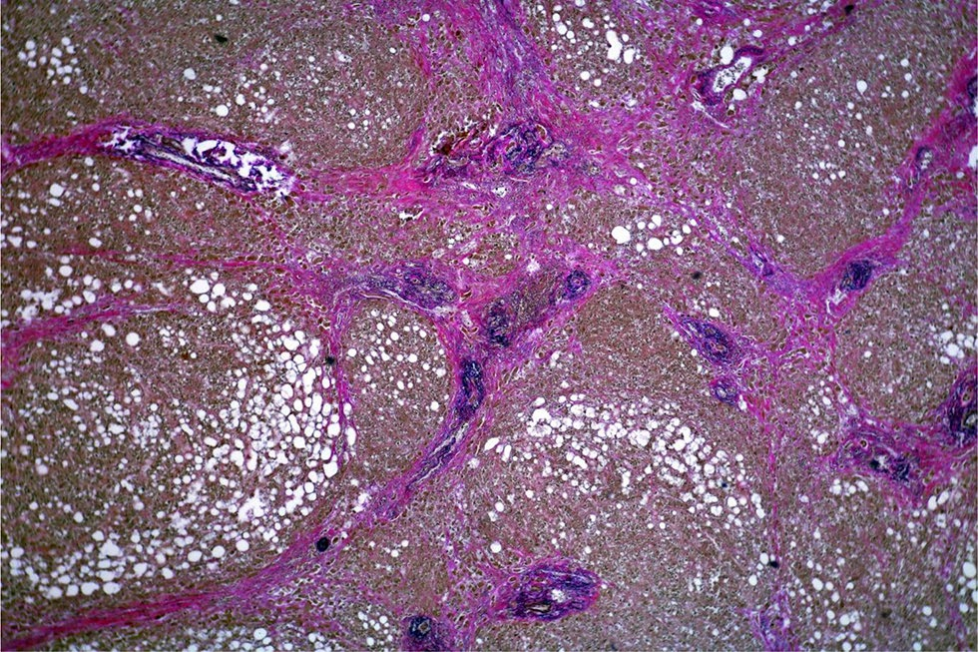
Fatty liver hepatitis with mixed dripping fatty degeneration and formation of periportal septa and incipient incomplete cirrhotic remodeling (EvG, magnification 40:1)

Analogous to the macroscopic impression, the liver showed mixed fatty degeneration with periportal septation and incomplete cirrhotic remodeling. The findings indicated fatty liver hepatitis (Fig. 4).

#### Skin and fistula

**Fig. 5:**
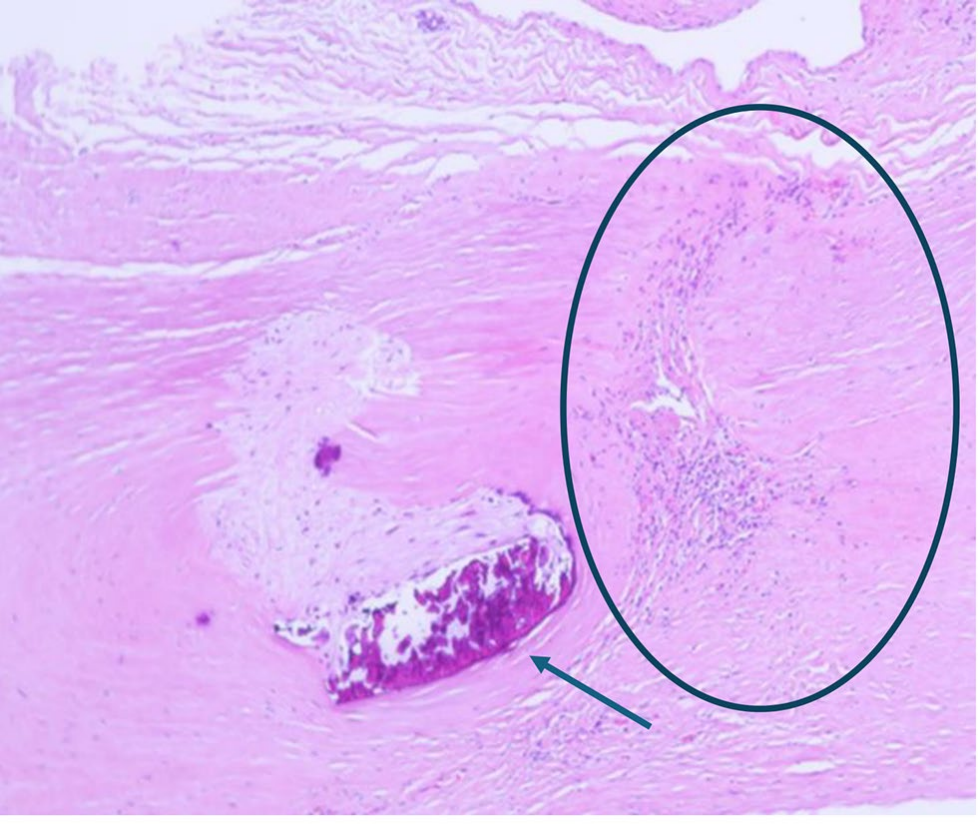
Vessel wall near the fistula site with incipient calcified scar (arrow), as well as another puncture site (circle) with incipient scarring, neovascularization and lymphocytes (HE, magnification 400:1); textural disturbance of the vessel wall (EvG, magnification 400:1)

**Fig. 6:**
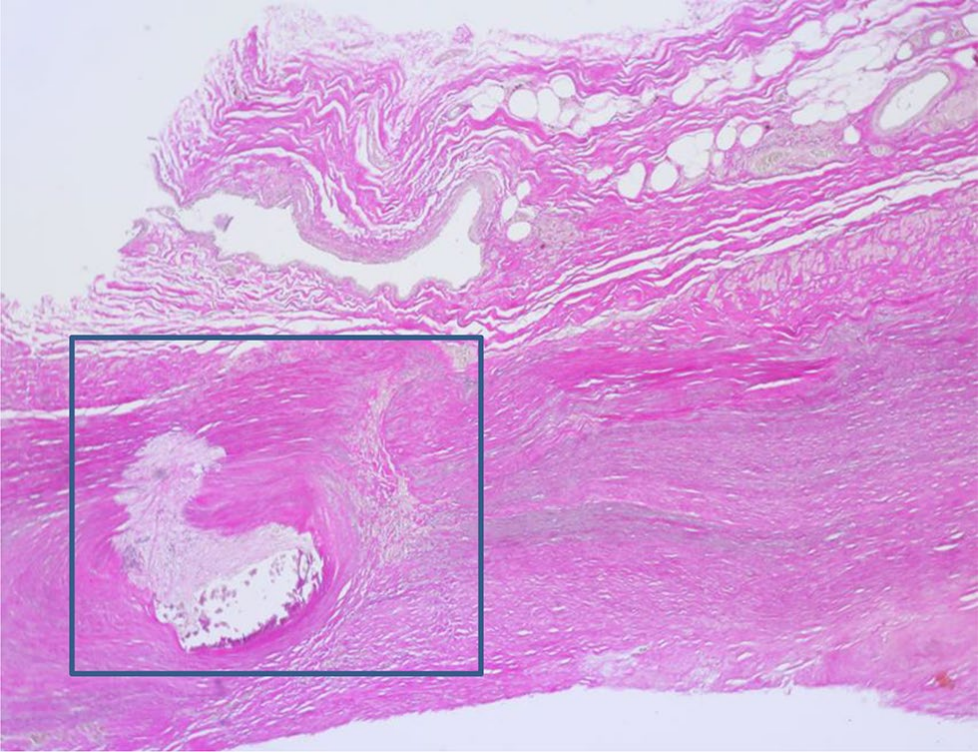
Textural disturbance of the vessel wall (EvG, magnification 400:1). The box corresponds to Fig. 5

The epidermis around the fistula showed reactive acanthosis with widening of the basal cell layer and regular keratinizing squamous epithelium. Numerous capillaries were visible within the upper dermis. Erythrocytes and fibrin deposits were found on the fistula itself. There were no severe chronic or acute inflammations of the skin and subcutis. The dermal connective tissue was partly scarred and partly spongily loosened. A zone with a slightly calcified scar and a puncture site with scarred transformation, neovascularization and lymphocytes was found near the vascular wall of the fistula site (Fig. 5). The elastic fibers were clearly rarefied in these parts of the vessel wall. In the parts of the vessel wall shown with scars/defect healing in the Elastica van Gieson staining, the elastic fibers showed a considerable texture disturbance in some areas (Fig. 6). Immunohistochemical examinations (CD 68, CD 45, SMA, glycophorin, CD 31) demonstrated that the hemorrhage in the vicinity of the fistula had occurred shortly before death [9].

## Discussion

In this presented case of the death of a senior dialysis patient, initially the possibility of an accident, suicide or medical malpractice as cause for the fatal hemorrhage [8, 10–13] were discussed. The forensic external post-mortem examination at the scene suggested a bleeding from a shunt vessel in the left upper arm as the cause of death. The cause of the bleeding initially remained unclear. The police investigation revealed no evidence of suicide, and the further forensic medical examination pointed towards the bleeding as a complication of dialysis treatment.

An autologous arteriovenous shunt (native AV fistula), as in the present case, is the lowest-risk and most common access route for long-term hemodialysis [4, 14]. Various techniques are possible for cannulation of the shunt vessel. In the rarely used “buttonhole technique”, the same puncture channel is punctured several times; in the more common “rope ladder technique”, the punctures are distributed over the shunt vessel, which reduces the risk of aneurysm, but requires access to a longer course of the vessel. In the equally common “area technique”, the punctures are distributed over an area of the shunt vessel that is only 2–3 cm long. It is used if the shunt vessel only has a short, sufficiently large lumen or otherwise runs too deep in the soft tissue. As a complication of the area technique, aneurysmal dilatation of the shunt can occur [14, 15]. Other shunt complications associated with long-term dialysis include infections, which can result in the risk of erosive bleeding and septic embolization [14]. In the present case, the “area technique” was performed for dialysis. The reasons given by the dialysis facility for this, i.e. the patient’s obesity and the deep course of the shunt vessel, were comprehensible during the autopsy. Corresponding to a typical complication, the shunt vessel was aneurysmatically dilated in the area of the punctures, and a fistula has formed between the shunt and the body surface.

Histology revealed no evidence of acute or chronic inflammation or necrosis of the tissue surrounding the shunt. However, textural disturbances were observed, with mainly missing elastic fibers in the vessel wall and the surrounding tissue as a result of repeated punctures over the years. Given this textural disturbance in the vessel wall and the adjacent soft tissue, leading to restrictions in the contractility of the vessel and thus to more severe bleeding [4]. Along with the reduction in retraction and healing capacity, the formation of a fistula and excessive bleeding appears to be a probable complicated outcome as the vessel’s ability to compensate for pressure differences is reduced due to a lack of elastic structures. Furthermore, the cirrhotic remodeling of the liver tissue might have led to a coagulation disorder that additionally aggravated the blood loss from the shunt vessel [16], where a blood flow of 400 to 2,000 ml/min may be present [4, 14]. Major blood loss was evident from the traces of blood in the apartment and the bloodstain pattern in the bathroom suggested bleeding from a high-pressure vessel.

Taking into account the general health situation of the woman, the situation in which she was found, the results of the investigation and the results of the autopsy and in particular the histological examinations, there were no indications of medical malpractice, and a complication of dialysis treatment was assumed as cause of the bleeding. As a partly rectangular-shaped bruise was present on the left upper arm, in the surrounding of the fistula, an additional blunt force trauma, e.g. caused by a bump, could be considered as an additional but not necessary trigger for the haemorrhage as the thin wall is particularly vulnerable to injuries [17].

## Conclusion

Bleeding from an arteriovenous dialysis shunt vessel is a forensically rare constellation in which many options regarding the triggering event must be considered. A detailed analysis of the case is essential in order to be able to answer the question of a possible suicide, accident or medical malpractice by taking a comprehensive view of the known pre-existing conditions, the circumstances at the scene of the event and the results of forensic examinations.

## Key points

1. Exsanguination from hemodialysis access site hemorrhages can occur for various reasons that may initially be unclear on external post-mortem examination.
2. A comprehensive review of the medical records and an assessment of the individual patient-related factors are essential for the accurate classification of the scenario.
3. In this case, histology provided valuable information about the cause of the hemorrhage, which was a typical complication of dialysis treatment.

## References

[1] Jager KJ, Kovesdy C, Langham R, Rosenberg M, Jha V, Zoccali C. A single number for advocacy and communication—worldwide more than 850 million individuals have kidney diseases. Kidney Int. 2019;96:1048–50. 10.1016/j.kint.2019.07.012. 31582227 10.1016/j.kint.2019.07.012

[2] Bello AK, Okpechi IG, Osman MA, Cho Y, Htay H, Jha V, Wainstein M, Johnson DW. Epidemiology of haemodialysis outcomes. Nat Rev Nephrol. 2022;18:378–95. 10.1038/s41581-022-00542-7. 35194215 10.1038/s41581-022-00542-7 PMC8862002

[3] Yeh L-M, Chiu SY-H, Lai P-C. The impact of Vascular Access types on Hemodialysis Patient Long-Term Survival. Sci Rep. 2019;9:10708. 10.1038/s41598-019-47065-z. 31341241 10.1038/s41598-019-47065-z PMC6656721

[4] Van Tricht I, De Wachter D, Tordoir J, Verdonck P. Hemodynamics and complications encountered with arteriovenous fistulas and grafts as vascular access for hemodialysis: a review. Ann Biomed Eng. 2005;33:1142–57. 10.1007/s10439-005-5367-X. 16175669 10.1007/s10439-005-5367-x

[5] Byard RW, James RA. Forensic issues in cases of fatal hemorrhage from arteriovenous dialysis access sites. Forens Sci Med Pathol. 2007;3:128–32. 10.1007/s12024-007-0003-8. 10.1007/s12024-007-0003-8 25869045

[6] Schmidt M-K. Tod durch Verbluten- unter besonderer Berücksichtigung ungewöhnlicher Todesfälle. Doctoral Thesis, Staats- und Universitätsbibliothek Hamburg Carl von Ossietzky; 2010. https://ediss.sub.uni-hamburg.de/handle/ediss/3749 (accessed December 17, 2023).

[7] Živković V, Cvetković D, Nikolić S. Exsanguination from arteriovenous fistula: anything is possible! Forensic Sci Med Pathol. 2020;16:381–2. 10.1007/s12024-020-00224-5. 32125632 10.1007/s12024-020-00224-5

[8] Kutschera L, Babian C, Tse R, Dreßler J, Ondruschka B. Exsanguination from iatrogenic puncture of arteriovenous fistula. Forensic Sci Med Pathol. 2020;16:379–80. 10.1007/s12024-019-00194-3. 31712985 10.1007/s12024-019-00194-3

[9] Betz P, Hausmann R, Wundaltersschätzung P. Rechtsmedizin. 2007;17:55–66. 10.1007/s00194-006-0418-7.

[10] Gill JR, Maloney KF, Hirsch CS. The consistency and advantage of therapeutic complication as a manner of death. Acad Forensic Pathol. 2012;2:176–81. 10.23907/2012.025.

[11] Gill JR, Goldfeder LB, Hirsch CS. Use of Therapeutic Complication as a manner of death. J Forensic Sci. 2006;51:1127–33. 10.1111/j.1556-4029.2006.00222.x. 17018093 10.1111/j.1556-4029.2006.00222.x

[12] Handlos P, Marecová K, Smatanová M, Dvořáček I, Dobiáš M. Fatal hemorrhage from an Arteriovenous Fistula. J Forensic Sci. 2018;63:1577–81. 10.1111/1556-4029.13730. 29341134 10.1111/1556-4029.13730

[13] Kölzer SC, Birngruber CG, Ramsthaler F, Kölzer JT, Verhoff MA. ungewöhnlicher verblutungstod im krankenhaus durch eine offene steckverbindung im infusionssystem. Anästh Intensivmed. 2019;60:457–62. 10.19224/ai2019.457.

[14] Geberth S, Nowack R. Praxis Der Dialyse. Berlin, Heidelberg: Springer; 2014. 10.1007/978-3-642-41208-0.

[15] Hepp W, Koch M, eds. Dialyseshunts, Springer Berlin Heidelberg; 2017. 10.1007/978-3-662-52699-6

[16] Fierro-Angulo OM, González-Regueiro JA, Pereira-García A, Ruiz-Margáin A, Solis-Huerta F. Macías-Rodríguez, hematological abnormalities in liver cirrhosis. World J Hepatol. 2024;16:1229–46. 10.4254/wjh.v16.i9.1229. 39351511 10.4254/wjh.v16.i9.1229 PMC11438588

[17] Schumpelick V, Bleese N, Mommsen U, editors. Kurzlehrbuch Chirurgie, 8., vollständig überarbeitete und erweiterte Auflage, Thieme, Stuttgart; 2010.

